# Design, testing, and scale-up of medical devices for global health: negative pressure wound therapy and non-surgical male circumcision in Rwanda

**DOI:** 10.1186/s12992-015-0101-4

**Published:** 2015-05-12

**Authors:** Gita N Mody, Vincent Mutabazi, Danielle R Zurovcik, Jean Paul Bitega, Sabin Nsanzimana, Sardis H Harward, Claire M Wagner, Cameron T Nutt, Agnes Binagwaho

**Affiliations:** Department of General Surgery, Center for Surgery and Public Health, Brigham and Women’s Hospital, Boston, MA USA; Rwanda Biomedical Center, Kigali, Rwanda; Worldwide Innovative Healthcare (WiCare), Cambridge, MA USA; Ministry of Defense of Rwanda, Kigali, Rwanda; The Dartmouth Center for Health Care Delivery Science, Hanover, NH USA; Global Health Delivery Partnership, Boston, MA USA; Ministry of Health of Rwanda, Kigali, Rwanda; Harvard Medical School, Boston, MA USA; Geisel School of Medicine at Dartmouth, Hanover, NH USA; Partners In Health, Boston, MA USA

Products with high efficacy and low cost are desirable in all market sectors and environments, particularly in settings where resources are limited. The health sectors of developing nations are an example of this basic economic principle as constrained financial and human resources must be budgeted toward large (and often, growing) populations’ health needs. However, the cost and quality characteristics that are absolutely necessary in resource-limited settings (RLS) remain highly desirable in wealthy markets as well. Consequently, technologies and strategies designed in RLS are frequently adopted by high-income nations, a process termed “reverse innovation” [1-4].

In recent years, some medical and surgical devices designed for RLS have been adopted by high-income nations. These reverse innovations have simultaneously overcome historical barriers to medical device deployment in RLS and challenged previously held assumptions regarding the direction of information transfer between high- and low-income nations. The potential for reverse innovation has subsequently been proposed as a reason in and of itself to develop products for RLS [4]. Products that result in reverse innovation offer improved care quality and treatment outcomes at lower costs to health care systems, expand markets for manufacturers, promote bidirectional transfer of information, and strengthen global partnerships for health equity [1-4].

One country that has made investments in myriad health innovations is Rwanda, a landlocked East African nation of approximately 12 million. Within the past two decades, Rwanda’s limited resources and diverse health care needs have combined to produce health care innovations ranging from community-based service delivery pathways to novel vaccine roll-out strategies [5-10]. Rwanda’s innovative approaches to seemingly insurmountable health challenges, and the nation’s resounding successes in these initiatives, have been described in a previous article in this *Globalization and Health* special series [2].

In the present article, we describe our experience with medical device innovation in Rwanda through two case studies, highlighting approaches taken to accelerate development and facilitate bidirectional flow of information. We also discuss ongoing challenges to progress in the field of health technology innovation for RLS. In sharing our experiences, we add our voices to the call for health technology innovation for low- and middle-income countries (LMICs).

## Case study: Wound-Pump

The Wound-Pump is a mechanically-powered simplified negative pressure wound therapy (sNPWT) device that applies a vacuum to a wound dressing. Initially conceived in 2007 and manufactured by WiCare Solutions, design features of the Wound-Pump address several inadequacies of other sNPWT systems for use in LMICs. Electrically powered Negative Pressure Wound Therapy (NPWT) systems have been proven to speed wound healing in diverse settings around the world, providing cleaner, less painful and more convenient hospital- and home-based wound treatment compared to conventional methods [11,12]. Non-healing wounds are ubiquitous, and create a clinical need for NPWT systems in developed and developing areas alike [13,14]. The authors’ clinical experiences through participation in long-standing partnerships between institutions based in Rwanda and the United States indicated the need for practical and affordable wound therapies in Rwanda. Although several existing NPWT systems provide the desired clinical results, cost and power supply have limited access to these therapies in RLS, including Rwanda [13,14].

The Wound-Pump was developed between 2007 and 2012 through a multidisciplinary design process in which the project engineer and clinical staff conducted extensive field testing in LMICs. The Wound-Pump has undergone testing to establish safety and biomechanical performance in Rwanda (ClinicalTrials.gov ID NCT01339429), and is currently undergoing clinical use approval process. Once manufacturing and distribution are completed, wound treatment with the Wound-Pump will be initiated in referral and district hospitals and subsequently maintained by community health workers at home and in health centers.

In addition to establishing safety and efficacy, however, the Wound-Pump design process addressed several challenges that have prevented market penetration by similar products. In particular, access to device materials and a dependable electrical supply have limited uptake of NPWT systems in many LMICs. Even in environments where the requisite materials and electrical supply are available, prohibitively high device costs may limit access to otherwise suitable technologies. Another non-electrical sNPWT system, SNaP, manufactured by Spiracur, encountered these challenges during trials in Tanzania: despite its ability to operate without electricity, limited distribution channels for its highly specialized, spring-powered components and high prices (180–200 USD/device) created obstacles to scaling up use of the system [15,16].

**Figure 1:**
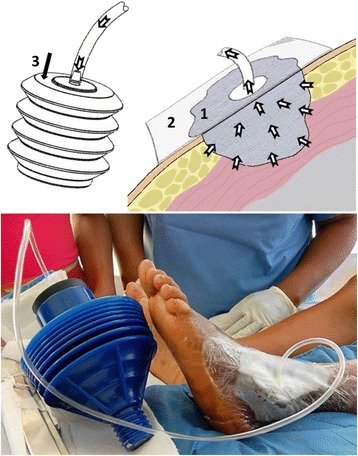
Mechanically powered Wound-Pump design.

In contrast to the SNaP system design process in the United States, development of the Wound-Pump system in LMICs allowed product assessment and modification in the environments in the settings in which the device will be deployed. Due to limited electrical supply in target regions, the Wound-Pump is mechanically powered, employing a bellows hand-pump is used to apply a vacuum to an airtight wound dressing (Figure 1) [14]. Unlike the SNaP system, however, the Wound-Pump system incorporates reusable materials that are or will be easy to obtain anywhere in the world. Furthermore, the Wound-Pump can be manufactured for less than 3 USD per device, drastically reducing consumer costs [14].

Field-based product testing also allowed early identification of potential device failures. The original Wound-Pump device prototype utilized a dressing design similar to that employed by other mechanically powered sNPWT systems [17], which was shown to be effective in maintaining suction with the bellows hand pump under laboratory conditions [13]. Our experience with the Wound-Pump in Haiti following the 2010 earthquake quickly demonstrated existing dressing designs to be ineffective on contoured body locations and in hot and humid climates when coupled with the bellows power source, however. A flexible polymer was therefore added to seal the dressing to the skin, the dressing was incrementally modified to address new issues identified by point of care clinical staff – such as patient immobility in external bone fixation devices and healthcare worker technical skill in applying dressings – over the course of clinical trials in Rwanda. Through this process, the dressing design was finalized within two years of initiating trials.

## Case study: PrePex male circumcision device

The PrePex device offers a non-surgical method for performing male circumcision (MC). MC is proven to be effective in reducing the lifetime risk of HIV infection by up 60% in males [18-22]; the United Nations Programme on HIV/AIDS (UNAIDS) has set a target of 20 million MCs by 2015 [23]. The PrePex device has met prequalification requirements of the World Health Organization (WHO) for use in the developing world [24], and has received approvals from the Conformité Européenne mark (CE mark), United States Food and Drug Administration (US FDA), and the International Organization for Standardization (ISO).

**Figure 2:**
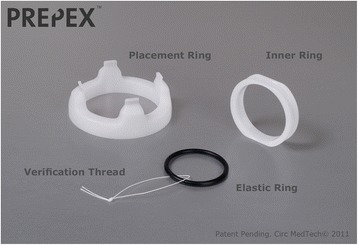
Components of the Prepex device.

The design of the PrePex device offers numerous advantages over traditional surgical MC in LMICs. Surgical MC is accomplished by cutting the foreskin and requires anesthesia, sterile environments and trained health care providers – all barriers to MC in the developing world [25]. The PrePex device, invented in Israel by Circ MedTech and subsequently trialed in Rwanda, involves concentric rigid and elastic rings that are positioned to cut circulation to the distal portions of the foreskin (Figure 2). The device remains in place for one week while the ischemic foreskin necroses, after which the device and the necrotic foreskin can be painlessly removed [26-28]. Furthermore, MC with PrePex is cost effective compared to surgical MC, especially in areas where national regulations allow the PrePex procedure to be performed by non-physician health care personnel [29,30]. The flexibility to perform MC with the PrePex device under non-sterile conditions expands the variety of settings in which the device may be deployed, and the brief procedure times (typically less than 5 minutes, including preparation) allow high patient throughput [27,28].

Field-based testing to establish the safety and efficacy of the PrePex device and the procedure for its clinical use occurred through a series of trials led by the Ministry of Health (MoH) of Rwanda [26-28]. These sequential studies adapted the PrePex device placement and removal procedures to minimize the complexity of the procedures, and optimize clinical outcomes and patient satisfaction. Since these initial studies, local PrePex experts have led training for professional use of the device in Rwanda; the MoH has trained 13 medical doctors and 48 nurses to use the PrePex device and is actively training more. Additional field-based research conducted in Rwanda also demonstrated the feasibility of device use by non-physician health care providers and acceptability of MC by PrePex to target populations [28]. The device is now being used in district hospitals and health centers as part of a comprehensive package of national HIV prevention strategies, and, through its use in campaigns, seems to have increased demand for MC among Rwandan youth [27,28]. The 2013 Rwanda HIV and AIDS National Strategic Plan has set a goal of providing voluntary MC to 700,000 males by 2015, and scaling up MC services is anticipated to play a key role in the national target of reducing new annual HIV infections by half by 2018 [31].

## Elements of successful reverse innovation

The Wound-Pump and the PrePex device represent two medical devices designed in and for LMICs with characteristics that make them appealing to health care systems in developed areas. These examples of medical device reverse innovation resulted from a combination of several successful elements in the approaches taken to design, develop and deploy the devices.

First and foremost, both the Wound-Pump and PrePex devices offer solutions to current gaps in health care delivery in target regions. In some cases, the health threats to be addressed by a device may already be identified in local and global health care goals and priorities; such was the case with the PrePex device, which has the potential to reduce HIV transmission and so achieve Millennium Development Goal 6 [23,32]. Data on lower profile conditions are often lacking, however. We therefore emphasize the value of clinicians’ professional experience as a key source for identifying highly morbid and mortal conditions affecting vulnerable populations. Especially when coupled with information sourced from published literature, firsthand experience in the clinical settings can contribute intimate knowledge of the needs and limitations of particular health care delivery environments.

**Figure 3:**
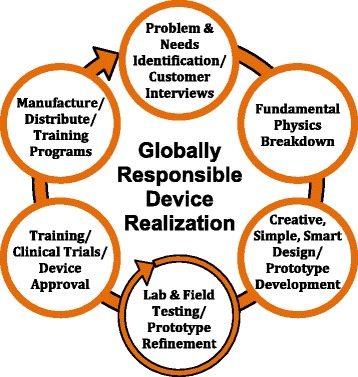
Globally responsible device designs require a deterministic process to translate clinical needs into an innovation that meets the requirements of the local environment.

Such knowledge is also indispensable during rigorous device design processes in which barriers to successful use of devices are addressed. Medical devices are often unavailable or non-functional in RLS due to lack of funds, materials, electrical power and maintenance capabilities [13,14,33]. Through a deterministic design process, clinical needs are deliberately broken down into a series of engineering tasks, and creative design solutions addressing existing barriers are developed (Figure 3). A device’s design itself may not be simple – device designs should achieve maximum impact according to globally recognized best practices – but technologies should also be unornamented and affordable in order to promote accessibility and adoption in LMICs [1]. Paramount to the design process is an emphasis on equal partnership with local clinicians and institutions where the device will be used. Exchange of feedback should be natural and welcomed, as many device developers actively practice and maintain teaching and training relationships in the clinical environments where the devices will be deployed.

Through such a process, key design issues encountered during the translation of promising ideas to point of care medical devices are addressed early and in a collaborative fashion. The deterministic design process is well illustrated by both the Wound-Pump and the PrePex device designs. In both cases, device modifications were made to accommodate human, financial and material resource limitations in target regions: the Wound-Pump is mechanically rather than electrically powered and contains reusable components, and the PrePex device can be employed without anesthesia or advanced surgical training.

Once local barriers to device use have been identified and addressed, field-based product testing is of critical importance to innovative device success. Prototype refinement can be accelerated by designing clinical trials to include stopping rules, embedded pilots, and quality checks. Field-based testing allows early detection of unanticipated complications in device deployment or performance, such as the ineffective dressing design originally employed in initial models of the Wound-Pump sNPWT system. Field-based testing also lends insight into the feasibility of device deployment in specific clinical environments and acceptability to potential patient populations, as was the case with early testing of the PrePex device in Rwanda.

Critical to the proper field testing of these devices is an in-country institutional review board with the authority to approve and terminate studies. In Rwanda, the National Ethics Committee is charged with review of all studies. At the conclusion of a study, results are mandatorily reported to the MoH and all participating partners. Budgets and timelines for device development must take these important steps, including additional unplanned testing phases, into account.

The final component of device innovation is seeing the device to market and use. Bidirectional partnerships and development of “local growth teams” are key strategies to achieving effective local implementation [34,35]. In our experience, device innovation in conjunction with local, field-based teams can serve as motivation for local health care providers by engaging their interest and creativity, thereby mitigating the drive behind the brain drain observed in LMICs [36,37]. Most importantly, truly sustainable implementation requires incorporation of the device into coordinated local health systems strengthening efforts, such as the Rwandan Human Resources for Health program [38].

## Challenges to medical device reverse innovation and scale up

Numerous challenges exist to scaling up use of novel medical devices. Sustainable funding for device implementation in LMICs remains an enormous challenge, especially for conditions neglected by global health funders, such as non-communicable diseases and injuries [1,39]. In-country manufacturing can keep distribution costs low and stimulate local economic growth in low-income settings, but for local manufacturing to be cost-effective it must be driven by appropriate infrastructure, accessible materials, supply chains, finances, and sustainable market demands and distribution channels.

Furthermore, international regulatory agencies maintain strict requirements to mitigate potential risks based on the class of a device; innovation of low-cost devices may be threatened by packaging requirements, shelf-life limitations, and other requirements imposed by regulators. While safety and high quality care must be maintained at all times throughout the testing and prototyping process, opportunities to adjust to local contexts have included the allowance of certain device components to be completely sterilized and reused. In understaffed health care facilities, family members often play a role in monitoring the patient once the patient is stable. Approval processes should take these contextually relevant considerations into account, and companies designing medical devices intended for use in LMICs should submit for global use approvals that account for the local realities that may affect device use.

## Conclusion

To the extent that reverse innovation may spur development of health technologies, it is likely to be an extremely positive force in the global health equity movement. Additionally, international industries may stand to benefit from low-margin, high volume models that enhance reach and access by selling larger quantities of less expensive devices to emerging markets via subsidiaries and in partnership with the local private sector or through public-private relationships. The resulting local economic opportunities could also stimulate local health care systems.

We urge the global health community and health technology industry to seek opportunities for novel medical device development to accompany global health efforts. There are many challenges inherent in this work, including limitations to infrastructure, materials, financing, market demands, and distribution channels, among others. But the process of navigating through and overcoming these challenges can often produce the most innovative solutions, and these solutions may be applicable in many settings. Ultimately, we believe global medical device innovation will improve access to care for all people, including the poorest and most disadvantaged, and we advocate for a defined and feasible pathway from need identification to device implementation and all the steps in between.
